# Anticancer potential of isoalantolactone in testicular cancer: an analysis of cytotoxicity, apoptosis, and signaling pathways

**DOI:** 10.18632/aging.206076

**Published:** 2024-10-09

**Authors:** Ming-Tse Sung, Hsuan-En Huang, Ya-Chuan Chang, Chia-Ying Yu, Hao-Lun Luo, Wen-Wei Sung

**Affiliations:** 1 Department of Pathology, Kaohsiung Chang Gung Memorial Hospital and Chang Gung University College of Medicine, Kaohsiung 83301, Taiwan; 2 School of Medicine, Chung Shan Medical University, Taichung 40201, Taiwan; 3 Department of Urology, Chung Shan Medical University Hospital, Taichung 40201, Taiwan; 4 Department of Urology, Kaohsiung Chang Gung Memorial Hospital and Chang Gung University College of Medicine, Kaohsiung 83301, Taiwan; 5 Center for Shockwave Medicine and Tissue Engineering, Kaohsiung Chang Gung Memorial Hospital and Chang Gung University College of Medicine, Kaohsiung 83301, Taiwan; 6 Institute of Medicine, Chung Shan Medical University, Taichung 40201, Taiwan

**Keywords:** testicular neoplasm, cell death, hypoxia inducible factor 1 subunit alpha, isoalantolactone, germ cell tumour

## Abstract

Testicular cancer, a highly prevalent malignancy among young adults, has witnessed an alarming rise in recent decades. This study delves into the therapeutic potential of isoalantolactone (IATL), a natural product extracted from Inula helenium and Inula racemosa, against testicular cancer. Employing MTT assays and flow cytometry, we observed a dose-dependent reduction in cell viability and induction of cell cycle arrest at sub-G1 phase with increasing IATL concentrations. Furthermore, Annexin V/PI dual staining revealed IATL-induced apoptosis. Human Apoptosis Array analysis demonstrated IATL’s influence on HIF-1α and TNF R1 expression, implicating its role in cancer cell growth and death regulation. Next-generation sequencing (NGS) and pathway analysis highlighted the involvement of ferroptosis and HIF-1 signaling in IATL-mediated effects. Western blotting validated the downregulation of key proteins associated with apoptosis inhibition and activation, confirming IATL’s potential as an anticancer agent. Moreover, IATL induced ferroptosis by modulating expression levels of GPX4, xCT, NRF2, and HO-1. Our findings shed light on IATL’s multifaceted anticancer mechanisms, emphasizing its potential as a therapeutic candidate for testicular cancer.

## INTRODUCTION

Testicular cancer is the most easily diagnosed tumor in patients 15–40 years old [1]. Based on Global Cancer Observatory (GLOBOCAN) data, there were 74,458 new cases in 2020, with a 5-year prevalence of all ages of 7.55 per 100,000. In addition, there were 9,910 new cases in the US in 2022 [2]. With a continuously increasing rate in some regions over several decades, testicular cancer has received much more attention, especially in Western and Northern Europe. The incidence of testicular cancer is approximately nine cases per 100,000 people, compared to less than one case per 100,000 people in Asia and Africa. Although the global occurrence rate of testicular cancer is relatively low, its prevalence among young people is extremely high. Thus, testicular cancer is still worth discussing and further researching [3–6].

Testicular cancer can be classified into germ cell tumors and non-germ cell tumors; among those, 95% of cases are associated with germ cell tumors. Additionally, germ cell tumors include pure seminoma and non-seminoma germ cell tumors (NSGCT) [7]. NSGCTs comprise four different histologic patterns: embryonal carcinoma, yolk sac carcinoma, choriocarcinoma, and teratoma, all of which have various behaviors. There are several clinical treatments, such as orchiectomy with/without retroperitoneal lymph node dissection, active surveillance, radiotherapy, and adjuvant chemotherapy with carboplatin or cisplatin [8]. In addition, chemotherapy drug resistance is getting more attention, and cancer cells may construct more efficient damage repair mechanisms to reduce the threat of chemotherapy drugs [9]. Therefore, this study about testicular cancer aims to develop a novel option for systemic therapy with less reproductive toxicity.

Isoalantolactone (IATL) is a natural product extracted from Inula helenium and Inula racemosa, serving as a medical plant in India and China [10]. IATL is known for its bio-function, such as anti-microbial, anti-inflammatory, anti-trypanosomal, and anti-proliferative [28]. Furthermore, IATL has multiple effects, including inducing detoxifying enzymes, regulating apoptosis and arresting the cell cycle [11–14]. IATL has anticancer potential in several cancer cell lines, such as pancreatic, prostate, colon, and lung carcinoma [15–17]. IATL functioned as a ROS-mediated apoptosis inducer by interacting with Bcl-2 protein families, such as Bcl-2 and Bcl-xl. In addition, IATL could regulate caspase proteins like Cas/c-Cas3, Cas/c-Cas7, and PARP/c-PARP, which activate apoptosis. Moreover, IATL inhibits constitutive NF-κB activation, and the NF-κB family inhibits apoptosis by inducing the expression of Bcl-2 family members and caspase inhibitors [18]. Although many signaling pathways have been related to IATL in treating multiple cancers, the underlying mechanisms of IATL in testicular cancer are still worth investigating.

Because of the issue of chemotherapy drug resistance and the fact that current treatments may cause sexual dysfunction and infertility, developing a new adjuvant therapy is essential [19]. IATL has shown anti-proliferative ability in other cancers and low toxicity to normal human cells [20]. These results offer a new perspective that IATL has potential in testicular cancer. This study investigated whether IATL can regulate the cell cycle and active apoptosis in testicular cancer cells.

## MATERIALS AND METHODS

### Cell culture

In our experiment, we chose NCCIT and NTERA2 as our cancer cells; both cause pluripotent embryonal carcinoma. Two testicular cancer cell lines were purchased from the Bioresource Collection and Research Center (Taiwan) and stored according to the suppliers’ brochure. NCCIT cells were maintained in RPMI 1640 medium, and NTERA2 cells were maintained in high-glucose (4.5 g/L) Dulbecco’s modified Eagle medium. 1 mM sodium pyruvate, 10% fetal bovine serum, 100 μg/mL of streptomycin, and 100 U/mL of penicillin were added to the medium. Two cell lines were cultivated at 37° C with a 5% CO_2_ atmosphere supplied [21, 22].

### MTT assay

An MTT assay was conducted to evaluate the cytotoxicity of IATL on NCCIT and NTERA2. In short, 10^4^/well testicular cancer cells were seeded in 96-well plates incubated overnight. Next, the cells were exposed to IATL (MedChemExpress, UK) at 0, 5, 10, 20, 40, and 80 μM for 24 hours. Next, we added an MTT solution (0.5 mg/mL) to the wells and incubated them at 37° C for three hours. The supernatant was then removed, and DMSO was added to dissolve the formazan crystals. An optical density of 570 nm was detected using an ELISA reader.

### Flow cytometry analysis

Flow cytometry was used to determine the cell cycle distribution and the percentage of apoptosis using the FACSCanto II binding buffer containing an I Cell Analyzer (BD Biosciences, San Jose, CA, USA). For cell cycle analysis, NCCIT and NTERA2 cells were treated with IATL (0, 5, 20 μM) for 48 hours. After being washed with phosphate-buffered saline (PBS), the cells were fixed in prechilled 70% ethanol at -20° C overnight. The fixed cells were then resuspended in PBS containing 0.5 mg/mL RNase A and 4 μg/mL PI (propidium iodide) to incubate at 37° C for 30 minutes in the dark. The suspensions were analyzed using flow cytometry. An Annexin V-FITC apoptosis detection kit (Strong Biotech Corporation, Taipei, Taiwan) was applied to analyze apoptosis based on the manufacturer’s protocol. NCCIT and NTERA2 cells were cultured in six-well plates with IATL (0, 5, 20 μM) for 48 hours. After harvesting, the cells were resuspended in 100 μL binding buffer containing 2 μL Annexin V-FITC and 2 μL PI. Following the 15-minute reaction in the dark, the cell apoptosis rate was evaluated using flow cytometry. The cell profiles were assessed using FlowJo software (BD Biosciences, Franklin Lakes, NJ, USA) [23, 24].

### Human Apoptosis Array for proteome profiling

The Human Apoptosis Proteome Profiler™ array (R&D Systems, Minneapolis, MN, USA) detected the expression of 35 apoptosis-related proteins. NCCIT and NTERA2 cells were treated with or without IATL (20 μM) for 24 hours, lysed, and 400 μg total protein was conducted for each array and analyzed based on the manufacturer’s protocol. Membranes with horseradish peroxidase-conjugated antibodies were presented using a chemiluminescent detection reagent. Then, we used ImageJ software to quantify the integrated density of the spots.

### Next-generation sequencing

NCCIT and NTERA2 cell lines were treated with IATL (0 and 20 μM) for 24 hours, and the total RNA was extracted with Trizol® Reagent (Invitrogen, Carlsbad, CA, USA) following the instructions provided in the manual. Genomics (Taiwan) conducted several processes, such as library preparation, sample preparation, sequencing, alignment, and differential expression analysis. All procedures were based on the official protocol. We conducted Gene Ontology (GO) and Kyoto Encyclopedia of Genes and Genomes (KEGG) enrichment analyses on the genes using the next-generation sequencing (NGS) database. The GO and KEGG pathways utilized the compare cluster function within the R package cluster profiler. The p-value of gene expression level ≤ 0.05 and ≥ 2-fold changes were considered significant differences. We visualized the genes with significant differences using a volcano plot, heat map, emap plot, etc.

### Protein extraction and western blotting

The NCCIT and NTERA2 cells were treated with IATL (0 and 20 μM; 24 hours) and washed twice with PBS. Then, a radioimmunoprecipitation assay (RIPA) buffer containing protease inhibitor and phosphatase inhibitor was added to the dishes. After centrifuging for 20 min at 10,400 rpm, the supernatant was saved at -80° C. 30 μg protein per sample was loaded into sodium dodecyl sulfate-polyacrylamide gels, then transferred onto a PVDF membrane (Millipore, Burlington, MA, USA). After blocking in 5% nonfat milk, the membranes were incubated with primary antibodies at 4° C overnight. The next day, the membranes were washed with Tris-buffered saline with 0.1% Tween 20 detergent (TBST) and incubated with secondary antibodies for one hour. In the end, the membranes were soaped in ImmobilonTM-Western Chemiluminescent HRP Substrate (Millipore, Burlington, MA, USA), and the results were presented using an ImageQuant LAS4000 instrument (GE Healthcare, Marlborough, MA, USA).

### Statistical analysis

The IBM Statistical Program for the Social Sciences (SPSS) software (version 20.0) was used to analyze the data. Statistics are presented as the mean ± SD. The student’s t-test was applied for continuous or discrete considered as statistically significant (* p < 0.05; ** p < 0.01; *** p < 0.001).

## RESULTS

### IATL exhibits cytotoxicity on testicular cancer cell lines and reducing cell viability

**Figure 1:**
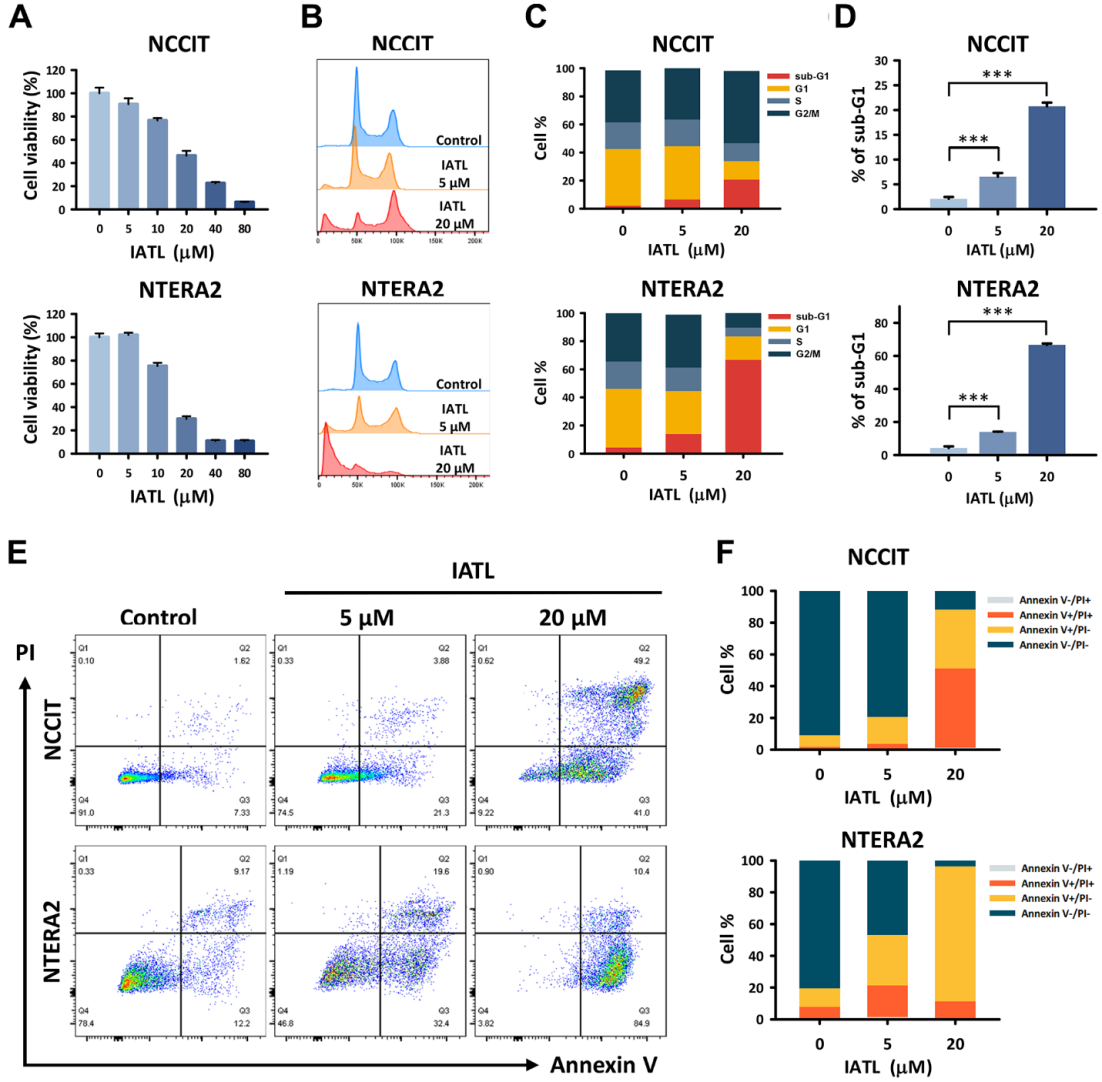
**IATL showed a cytotoxic effect and induced apoptosis in the NCCIT and NTERA2 cell lines.** (**A**) We conducted an MTT assay to analyze the cell viability in NCCIT and NTERA2 cell lines treated with IATL (0, 5, 10, 20, 40, and 80 μM) for 24 hours. In addition, flow cytometry was used to detect the cell cycle of testicular cancer cell lines after IATL treatment (0, 5, and 20 μM) for 48 hours. (**B**) The peak chart shows the cell accumulation in each phase; we defined 50k as a haploid chromosome. (**C**) The percentage of various phases in the cell cycle is shown in bar charts. (**D**) There are increasing trends of sub-G1 on both NCCIT and NTERA2 cell lines exposed to IATL. The data are shown as mean ± SD (** p < 0.01; *** p < 0.001). (**E**) Additional flow cytometry with Annexin V/PI dual staining was conducted to detect the stage of apoptosis. NCCIT and NTERA2 were treated with IATL (0, 5, and 20 μM) for 48 hours. (**F**) The bar chart shows the proportion of testicular cancer cells; the viable cells decreased after IATL treatment.

As mentioned above, IATL has shown cytotoxic effects on multiple cancers. Therefore, we verified its ability to suppress both NCCIT and NTERA2 cell lines. As shown in Figure 1A, NCCIT and NTERA2 were treated with IATL at different doses. The MTT assay revealed that the higher concentrations of IATL led to a more significant reduction in cell viability. Furthermore, we treated NCCIT and NTERA2 cells with IATL (0, 5, and 20 μM) for 48 hours and utilized flow cytometry to analyze the cell cycle progression (Figure 1B–1D). Figure 1B shows a peak chart demonstrating cell accumulation at distinct stages. We defined 50k as a haploid chromosome, 100k as a diploid chromosome, and testicular cancer.

Next, we determined that as the IATL concentration rose, more testicular cancer cells accumulated in the sub-G1 phase. The percentage of different phases in the cell cycle is shown in Figure 1C; the results also verify the relationship between IATL concentration and the cell cycle stage. As shown in Figure 1D, the result of NCCIT exposed to IATL was arrested in sub-G1 in a concentration-variant mode, from 2.10 ± 0.36% to 20.77 ± 0.76% NCCIT: control vs. 5 and 20 μM, p = 0.001). We found a similar result of NTERA2 treated with the same IATL concentration in sub-G1 (Figure 1D), from 4.28 ± 0.95% to 66.73 ± 0.76% (NTERA2: control vs. 5 and 20 μM, p = 0.045 and 0.001, respectively). Based on these results, IATL could inhibit cell mitosis and play an important role in testicular cancer cell apoptosis.

### IATL induces apoptosis in testicular cancer cell lines

To better understand the relationship between testicular cancer cell death and IATL, we conducted flow cytometry to detect apoptotic cells using Annexin V/PI dual staining (Figure 1E). After adding the IATL, more NCCIT cells presented on the first quadrant of the staining pattern, which meant cells stayed at the late stage of apoptosis. As for NTERA2, the most induced cells presented on the fourth quadrant of the staining pattern. In other words, the cells exposed to IATL tended to stay in the early stage of apoptosis. In Figure 1F, the columns signify the percentage of early and late apoptotic cells for NCCIT and NTERA2. For NCCIT cell lines exposed to IATL for 48 hours, the rates of apoptotic cells were from 8.86 ± 0.59% to 87.10 ± 3.44% (NCCIT: control vs. 5 and 20 μM, p = 0.045 and 0.001, respectively). The results of NTERA2 also presented a similar trend; the rates ranged from 19.11 ± 3.01% to 95.57 ± 0.55% (NTERA2: control vs. 5 and 20 μM, p = 0.001).

### Utilizing Human Apoptosis Array and next-generation sequencing to investigate IATL-related pathways in testicular cancer

The Human Apoptosis Array was used to analyze the signaling proteins related to INTL-induced apoptosis pathways. According to Supplementary Figure 1A–1D, IATL inhibits HIF-1α expression in NCCIT cell lines and regulates TNF R1 expression in NTERA2. HIF-1α promotes cancer cell growth and angiogenesis via hypoxic or non-hypoxic pathways, and TNF R1 is involved in TNF signaling pathways, which regulate cell death. We observed the upregulation of c-capspase-3 and downregulation of survivin in both cell lines. Based on the results of detecting 35 apoptosis-related proteins, it can be inferred that IATL is associated with HIF-1α signaling pathways and TNF signaling pathways, triggering downstream signal pathways that affect testicular cancer cell survival.

**Figure 2:**
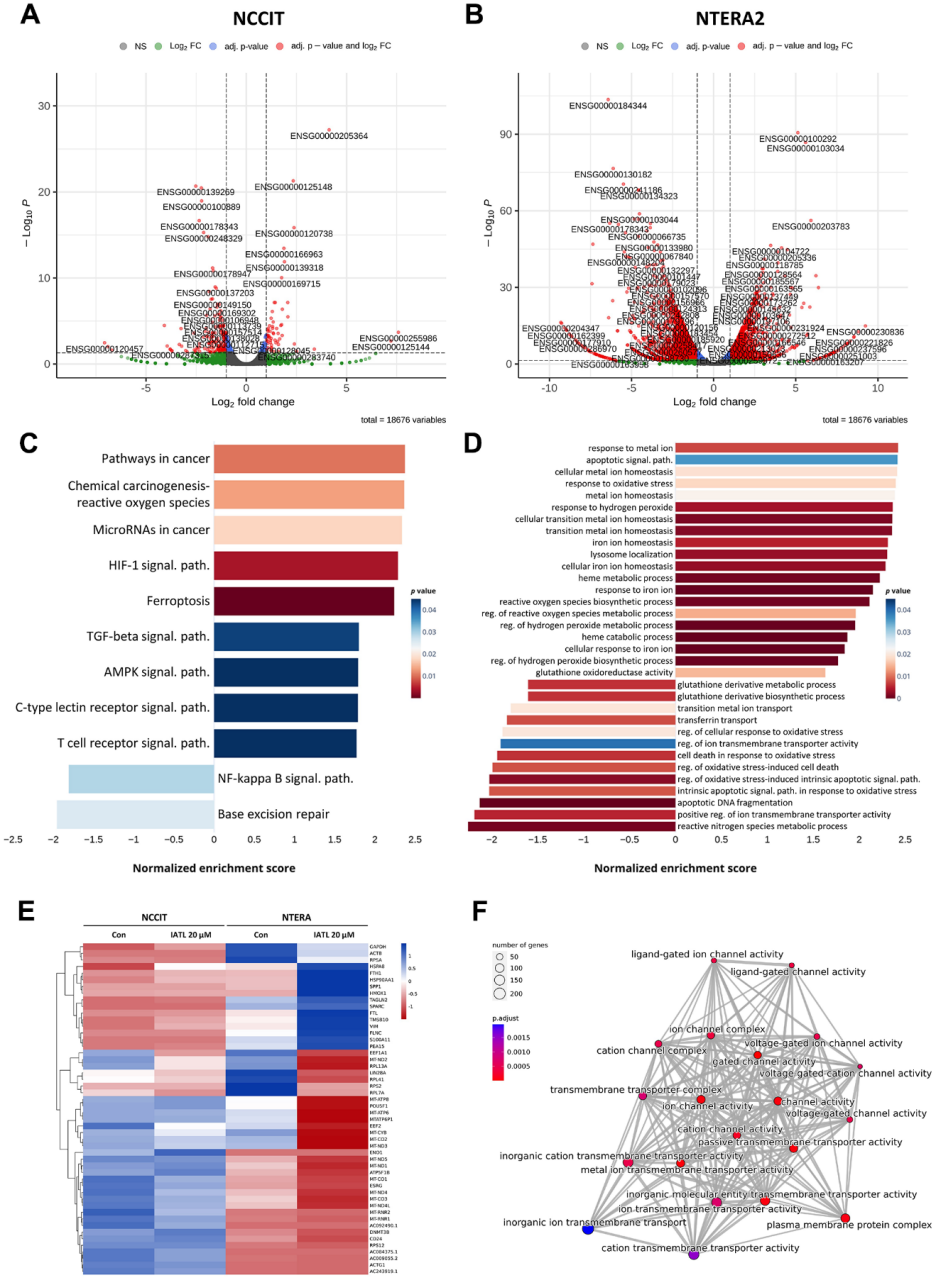
**KEGG and gene ontology (GO) enrichment analysis in IATL-treated NCCIT and NTERA2 cell lines.** (**A**, **B**) The volcano plots of NCCIT and NTERA2 visualized genes with significant differences in expression levels. (**C**) The KEGG enrichment analysis bar plot indicated the IATL-related pathways, which presented significant differences. (**D**) The bar plot of a GO analysis shows the cell cycle or apoptosis-related pathways, which changed expression level obviously after the NCCIT and NTERA2 were treated with IATL. (**E**) The heat map of the NGS database comparing the expression levels between the control group and the cell lines treated with IATL 20 μM. Blue: upregulation. Red: downregulation. (**F**) The emap plot (p.adjust < 0.05) showed the relationship between ferroptosis-related genes.

NGS was conducted to detect the IATL-related pathways in testicular cancer. Based on Figures 2A, 2B, the NCCIT and NTERA2 volcano plots illustrate significant gene differences. The normalized enrichment score (NES) displays the correlation coefficients between the different pathways and IATL, and the color of the bars indicates the p-value. Figure 2C shows the results of the analysis from the KEGG pathway database. We realized that ferroptosis and the HIF-1 signal pathway are the most related to the IATL mechanism in testicular cancer. Furthermore, we conducted an additional NGS analysis from the Gene Ontology (GO) database. GO can categorize each gene into three major aspects: cellular components, biological processes, and molecular functions. We visualized the results related to the biological process in Figure 2D. The figure shows that the response to iron ion, heme catabolic process, and regression of the hydrogen peroxide metabolic process represented a low p-value, which meant these results were statistically significant.

Additionally, the processes that exhibited significant differences in Figure 2D were associated with ferroptosis and the HIF-1 signal pathway. In Figure 2E, the genetic differences in the KEGG enrichment analysis were transformed into a heat map, and the horizontal axis represents the Z score. This demonstrated that NTERA2 showed a greater variance in genetic expression than NCCIT. To further investigate the relationship between IATL and ferroptosis, we explored the correlations between ferroptosis-related pathways. As shown in Figure 2F, there was a high degree of correlation among these pathways, such as the ion channel complex and the transmembrane transport complex. Thus, the results further verify that ferroptosis and HIF-1 signal pathways are IATL-related pathways in testicular cancer.

### Further investigation of the IATL-related apoptosis pathways

The NGS data gave us a new perspective to investigate the mechanism of IATL in testicular cell lines. We conducted a western blot to verify the hypothesis of the IATL-related apoptosis pathways.

**Figure 3:**
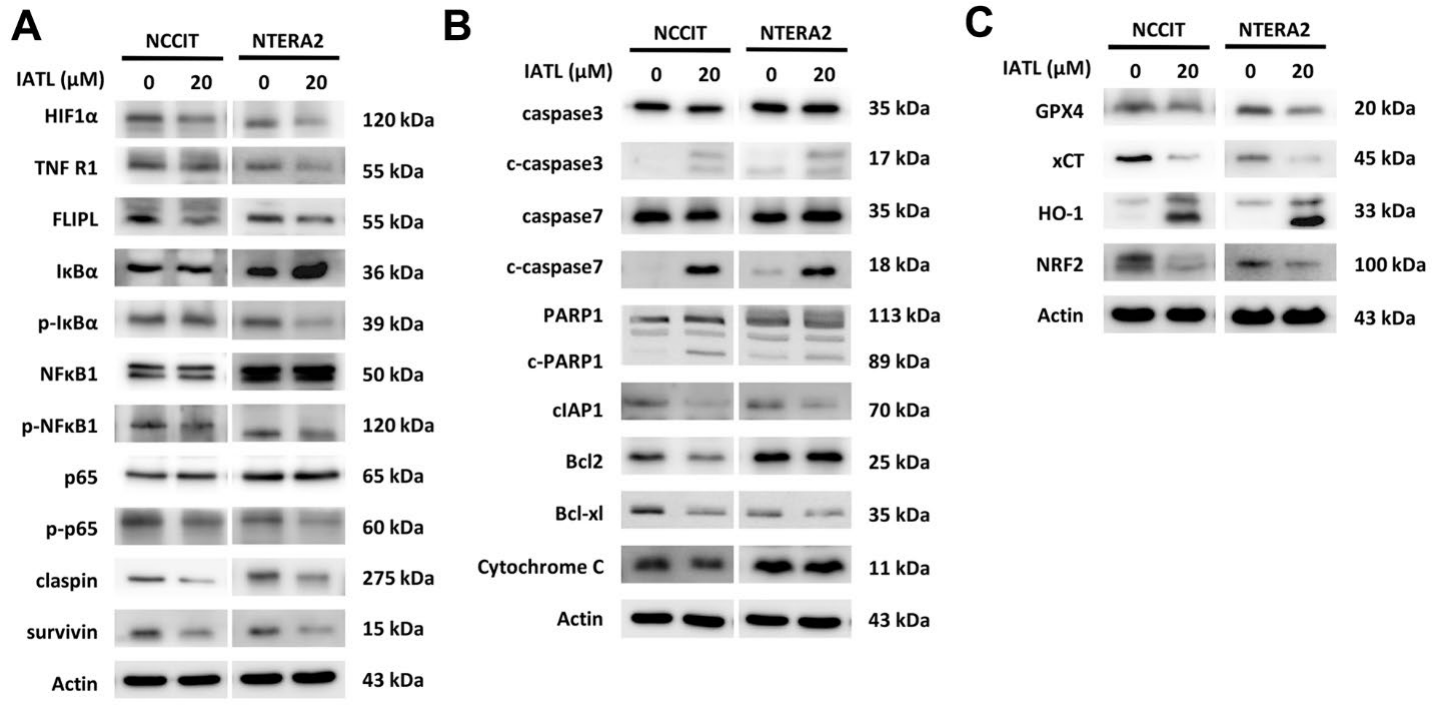
**IATL triggered apoptosis and ferroptosis pathways in testicular cancer cells.** (**A**) The western blot implied the decreasing expression of p-IκBα, p-NFκBα1 and p65, the downstream molecules of the TNFR1 pathway. (**B**) The apoptosis-related proteins presented the expected trends after IATL treatment. Our hypothesis that IATL could induce apoptosis in testicular cancer cell lines has been confirmed. (**C**) GPX4, xCT, and NRF2 demonstrated declining trends, and HO-1 presented an increasing trend. The results verified that IATL is related to ferroptosis.

Figure 3A shows that the expression level of HIF1α decreases after IATL administration. Furthermore, the downregulations of p-IκBα and p-NFκBα1 are apparent, and p-p65, a vital member of the NFκB complex, presented lower expression after testicular cancer cells were exposed to IATL. In addition, the downstream signal molecules, claspin and survivin, showed decreasing trends. According to Figure 3B, the upregulations of c-caspase 3 and c-caspase 7 implied that the apoptosis had been executed, and caspase 3 promoted PARP1 cleavage. Notably, cIAP1 and Bcl-xl both played essential roles in apoptosis inhibition, and their decreasing expression represented the potential anticancer effect of IATL.

In addition, we determined that IATL can trigger ferroptosis in testicular cell lines. Both GPX4 and xCT can prevent lipid peroxidation in the cell membrane. The excessive activation of HO-1 can induce the accumulation of ferrous ions, and NRF2 can regulate iron metabolism and initiate cellular anti-oxidation. In Figure 3C, GPX4, xCT, and NRF2 show decreasing trends, and HO-1 shows an obvious increasing trend. Based on this result, IATL can induce ferroptosis and alter the expression level of ferroptosis-related proteins.

## DISCUSSION

**Figure 4:**
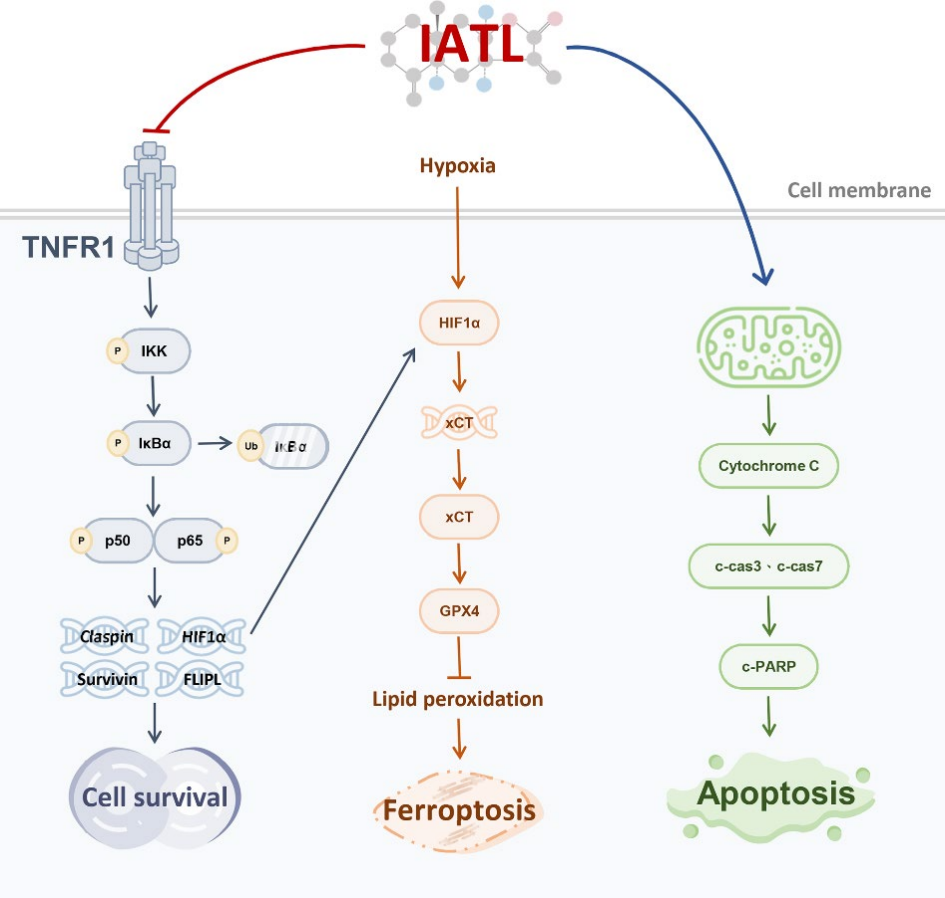
Schematic figure showing that IATL inhibits survival and promotes ferroptosis and apoptosis in testicular cancer cells via the TNF R1-induced NF-κB pathway.

Our study revealed that IATL inhibits testicular cancer cell survival and promotes testicular cancer cell ferroptosis, and apoptosis via the TNF R1-induced NF-κB pathway (Figure 4). Although both the mortality rate and prevalence rate of testicular cancer are low, the side effects of current therapies seriously impact patients’ lives. For example, radiation therapy or chemotherapy may cause a higher chance of hypertension and cardiovascular disease. Furthermore, patients who receive radiation therapy or chemotherapy can more easily develop infertility than do people who undergo orchiectomy alone. Therefore, we must explore the potential anticancer effects of IATL in testicular cancer cell lines [25, 26].

In ancient India and China, IATL was used as an herbal remedy for anti-microbial, anti-inflammatory, and anti-proliferative activities [27]. To date, there have been several studies about the anticancer effect of IATL on liver, breast, colorectal, pancreatic, and prostate cancer. However, the effects of IATL on testicular cancer cells have not been addressed. In addition to inducing apoptosis via the overexpression of ROS, IATL inhibits various tumor growth by different pathways. IATL-induced cell cycle arrest, apoptosis, and autophagy in multiple cancers, such as colorectal, breast, and pancreatic cancers. IATL suppressed AKT/mTOR signaling pathway in colorectal cancer, regulated breast cancer cell proliferation via MAPK/NF-κB signaling pathway, and induced pancreatic cancer cell apoptosis by PI3K and Wnt signal pathway [16, 28–31].

Our study chose NCCIT and NTERA2 as testicular cancer cell lines. NCCIT is a pluripotent stem cell line that may develop into testicular embryonal carcinoma and teratocarcinoma. NTERA2 is an epithelial-like cell line that also develops into testicular embryonal carcinoma. We concluded that IATL induced the G1/S cell cycle arrest and apoptosis and regulated the TNF R1-induced NF-κB and caspase cascade pathways.

Following NGS, we observed significant differences in the expression levels of the ferroptosis-related and HIF-1-related pathways. Distinct from apoptosis, ferroptosis is regulated cell death resulting from iron-dependent accumulation and lipid peroxidation. HIF-1 is a transcription factor for changes in cellular oxygen levels. HIF-1 is associated with iron metabolism and influences the uptake and storage of iron. In addition, HIF-1 can enhance the transcription of SLC1A1, thereby promoting ferroptosis resistance. We utilized GO and KEGG enrichment analysis to demonstrate that IATL can induce cancer cell death by regulating the expression of HIF-1 and ferroptosis-related pathways [32–34].

We conducted a western blot to verify that the downregulation of TNF-R1 reduced the expression of FLIPL, p-IκBα, p-NFκBα1, and p-p65. The reduced amount of claspin, presented at the S phase, also demonstrated that testicular cancer cells exposed to IATL tended to accumulate at the sub-G1 phase. Caspases play central roles in cell apoptosis, and caspase cascade apoptosis has been activated after IATL triggered the TNF-R1-induced NF-κB pathway. The increasing expression of c-caspase 3, c-caspase 7, and c-PARP implied that apoptosis had been executed. In addition, survivin, cIAP1, and Bcl-xl, anti-apoptosis proteins, are inhibited by IATL. Finally, we learned that IATL is associated with HIF1 and ferroptosis via GO and KEGG enrichment analysis. The western blot verified that IATL can alter the expression level of HIF1 and ferroptosis-related proteins. The results provided a new perspective that IATL can induce apoptosis and trigger ferroptosis in testicular cell lines.

The Bcl-2 family of proteins has traditionally been regarded as regulators of apoptosis, but recent studies have linked it to cellular senescence. Overexpression of Bcl-2 not only leads to cell cycle arrest and inhibition of apoptosis but also triggers cellular senescence. Our western blot results show a distinct difference in Bcl-2 protein levels between NCCIT and NTERA2 cells. Following treatment with IATL, Bcl-2 levels decreased in NCCIT cell lines, whereas there was no significant change in NTERA2. We speculate that the different cellular origins of NCCIT and NTERA2 might contribute to these variations in Bcl-2 expression. However, further research is necessary to fully understand the mechanisms underlying these differences [35].

Recent research indicates that KRAS copy number gain is highly prevalent in testicular cancer cells. However, the resistance of tumors to KRAS inhibitors remains a significant challenge in clinical treatment. It is known that KRAS can activate downstream pathways, such as the PI3K/AKT and NF-κB signaling pathways, which are also influenced by IATL. The potential anticancer effects of combining KRAS and IATL merit further discussion [36–38]. In addition, NRF2 regulates glycolysis and glutaminolysis in cancer cell lines. Jaemoo Chun demonstrated that IATL can suppress glycolysis in ovarian cancer cells. In our experiments, we determined that IATL influences the NRF2/HO-1 pathway to induce ferroptosis. Further research is needed to explore whether IATL can affect testicular cell metabolism by altering NRF2 expression [37–39].

Cisplatin is a common clinical drug against testicular cancer, but it exhibits significant nephrotoxicity in the human body. In addition, cancer cells may develop more efficient DNA repair mechanisms and a more extensive variety of cell membrane transport systems. Thus, chemotherapy drug resistance has become a significant issue [9, 40]. Compared to the current anticancer drug, IATL displays selective cytotoxicity on tumor cells from normal cells. Huang et al. indicated that the IATL and cisplatin combination can enhance the sensitivity to prostate cancer cells [15]. Although the IATL-induced pathways of multiple cancers differ, IATL is still a potential anticancer drug.

Numerous studies have explored the anticancer mechanisms of IATL across various cancers. The MEK/ERK signaling pathway is a crucial mediator for IATL in inhibiting gallbladder cancer progression. IATL increases the Bax/Bcl-2 ratio, mediates the mitochondrial pathway, and suppresses the MAPK/NF-κB signaling pathway to induce apoptosis in breast cancer cells. In colorectal cancer, the AKT/mTOR pathway acts as a negative regulator of autophagy, and IATL reduces associated protein levels to induce cell death and autophagy. For prostate cancer, IATL downregulates survivin protein expression and regulates ROS-dependent apoptosis. Furthermore, IATL enhances the sensitivity of prostate cancer to cisplatin-based treatments by cooperating with cisplatin to increase ER stress and activate the JNK signaling pathway [16, 28–30, 41].

Although the anticancer mechanism of IATL in other cancers has been widely discussed, this study is the first to discuss the anticancer effect of IATL on testicular cancer cells, so we still have a long way to go before it can be applied in clinical treatment. First, only NCCIT and NTERA2, which are related to embryonal carcinoma and teratocarcinoma, were chosen for the experiments. However, there are many complicated types of testicular cancer, and the efficacy of IATL against several types of testicular cancer cells requires further investigation. Secondly, cellular senescence acts as a natural barrier to tumorigenesis; therefore, it is necessary to conduct senescence analysis in future studies [42]. Third, after identifying the underlying mechanism of IATL-related pathways, *in vivo* experiments were essential to clarify its safety and effectiveness. If IATL successfully demonstrates an anticancer effect *in vivo*, we should consider the feasibility of IATL and other chemotherapy drug combinations. It is essential to resolve drug resistance and the side effects caused by current treatment strategies. Despite these limitations, this study offers a broader perspective on adjuvant treatments for testicular cancer.

## Supplementary Material

Supplementary Figure 1
