# Supplementary material for: Anticancer potential of isoalantolactone in testicular cancer: an analysis of cytotoxicity, apoptosis, and signaling pathways
Source: Aging (Albany NY). 2024 Oct 9;16(19):12820–32. doi: 10.18632/aging.206076 (PMC11501383; doi:10.18632/aging.206076)
Supplement: Supplementary Figure 1 [file aging-16-206076-s001.pdf]

SUPPLEMENTARY FIGURE

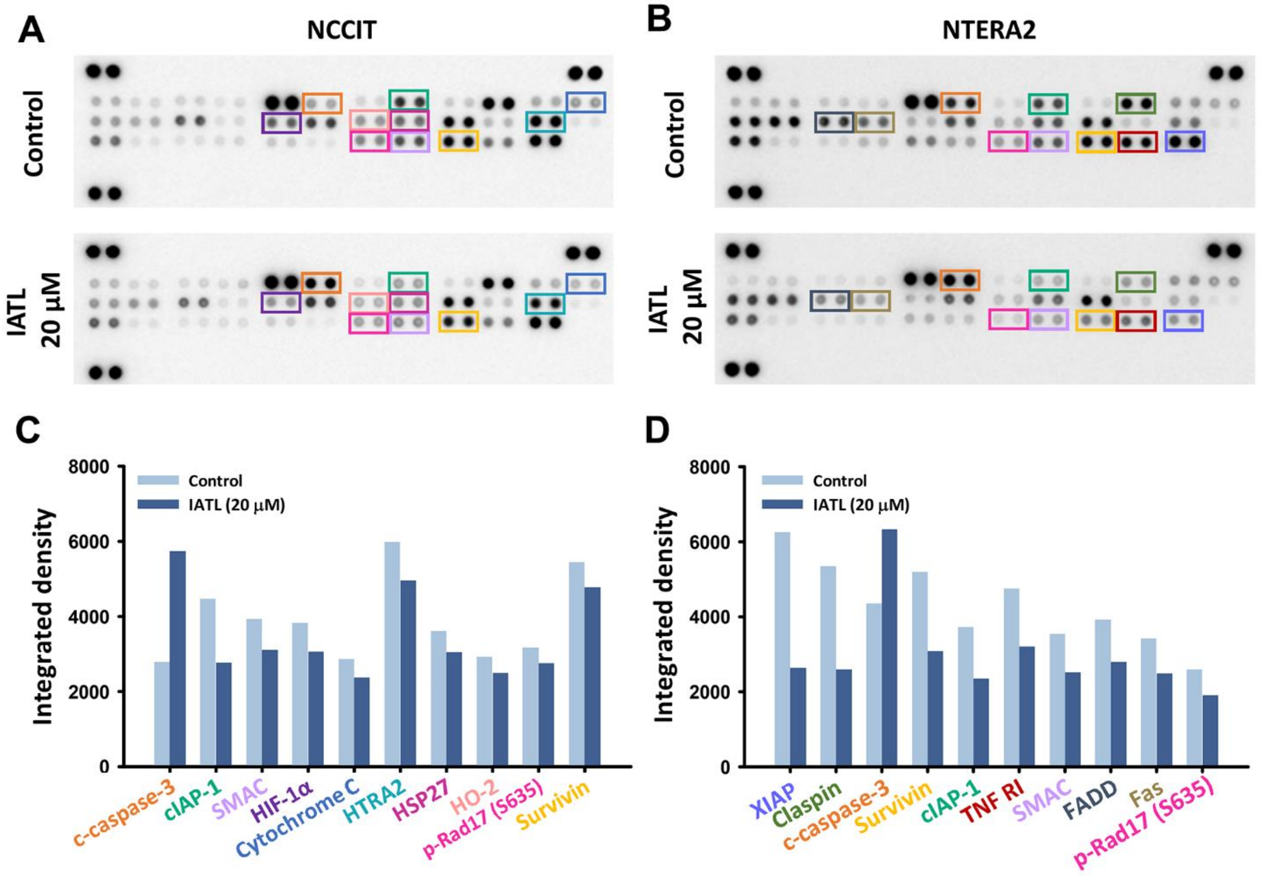

Supplementary Figure 1. We used the Human Apoptosis Array to analyze testicular cancer cell death caused by IATL. (**A**) NCCIT and (**C**) NTERA2 were examined for the expression of apoptosis-related proteins. As demonstrated in (**B**, **D**), the integrated density of proteins is quantified into bar charts; the proteins that presented obvious changes after IATL treatment gave us clues to determine the IATL-related apoptosis pathways.
